# Biomechanical response of lower limb joints to lateral wedge insoles

**DOI:** 10.1038/s41598-023-50693-1

**Published:** 2024-01-02

**Authors:** Weijin Du, Yuan Guo, Chenyan Wang, Weiling Cui, Weiyi Chen, Xiaona Li

**Affiliations:** College of Biomedical Engineering, Taiyuan University of Technology, Taiyuan, 030024 China

**Keywords:** Biomedical engineering, Disease prevention

## Abstract

Lateral wedge insole (LWI) is a frequently recommended treatment option for early and midterm stages of medial knee osteoarthritis. However, studies of its effects on the lower limb joints are incomplete and imperfect. The main purpose of this study was to quantitatively analyze the response of intervention of LWI on lower-limb joint kinematics, ground reaction forces (GRFs), and centre of pressure (COP). Gait analysis of 16 healthy subjects was conducted. Three-dimensional motion data and force plate measurements were collected in the control (barefoot) and experimental conditions (wearing a pair of assigned shoes with 0, 7, and 10 mm LWIs). Results showed that the peak knee flexion angle was increased by 3.43°, 3.09°, and 3.27° with 0, 7, and 10 mm LWIs, respectively (*p* < 0.01). The ankle peak dorsiflexion angle was significantly decreased by 3.79°, 2.19°, and 1.66° with 0, 7, and 10 mm LWIs, respectively (*p* = 0.02). The internal rotation angle was increased by 2.78°, 3.76°, and 4.58° with 0, 7, and 10 mm LWIs, respectively (*p* < 0.01). The forefoot with LWIs showed highly significantly smaller inversion, eversion, and adduction angles (all *p* < 0.01). The 1st peak of the vertical GRF (*p* = 0.016) also increased significantly by a maximum of 0.06 body weight (BW) with LWIs. These results indicated that biomechanical changes and limitations of lateral wedges insole should be analyzed in more detail, possibly leading to new guidelines for the design and application.

## Introduction

The lower limb structure is an extremely important part of the human body, which is involved in basic movement functions such as standing, sitting, walking and stair climbing in daily life. As the end joint of the limb, the foot, which is a complex mechanical structure consisting of 26 bones, 33 joints, 107 ligaments, 19 muscles and other connective tissues, bears the body weight and allows locomotion^1^. Insoles are one of the interventions with the most direct connection and impact on the foot, which may directly change the mechanical axis of the lower limb^2^ and even trigger a chain of deformation and degeneration of the lower limb joints and even the spine^3^. In recent years, orthopaedic insoles have been used as a non-invasive economical adjunctive treatment method for patients with osteoarthritis of the knee^4^, foot pathologies^5^ and flatfoot^6^. Among these, the lateral wedge insole (LWI) has become a research hotspot regarding its effectiveness in the early and midterm stages of medial knee osteoarthritis (KOA).

The LWI, placed inside shoes, is a non-invasive economical treatment that decreases medial tibial plateau loads by altering the external knee adduction moment (KAM) during walking^7–9^. The KAM is determined by a combination of the ground reaction forces (GRFs) and the GRF to knee centre lever arm. The LWI shifted the calcaneus to the valgus position relative to the tibia, shifted the foot centre of pressure (COP) and GRF laterally, and produced a more vertical GRF. This caused the femur to become more vertical and undergo more adduction than before, thereby reducing the lever arm from the GRF to the knee centre and the knee valgus angle, leading to a reduction of KAM^10^. The effect of wearing LWIs on osteoarthritic patients with a varus deformity of the knee was first studied in the 1980s^2^. It was shown that the LWI triggered a change in the spatial position of the lower limb. In the following decades, the response of external KAM to LWI has been researched by many scholars and the external KAM is gradually becoming an accepted indirect measure of medial compartment loading of the knee^11^. Relevant studies showed that LWI could reduce the peak external KAM by about 5% on the treated side, but they were not necessarily effective in relieving knee pain^7,12–15^. The GRFs and COP are important factors affecting KAM, so it is necessary to study the influence of LWI on them. The lateral wedge height is an important indicator which contributes to foot valgus when customizing the LWI. It represents the height difference between the lateral and medial heights at the metatarsal of the insole. Several researchers have reported on the effect of lateral wedge height on lower limbs biomechanical variables^16^, spatiotemporal parameters^17^, and comfort^18^ in patients with knee osteoarthritis. Studies have suggested that the foot valgus of more than 8° can cause discomfort for the patient. However, few have evaluated the relationship between these biomechanical parameters and the wedge height.

Furthermore, much more attention was paid to investigating whether the LWI was indeed effective in improving knee osteoarthritis. There were fewer concerns about its impact on other associated lower extremity structures, particularly the foot and ankle. Recently, growing clinical data has revealed that numerous patients with KOA were often accompanied by foot or ankle disease, which has led to an increasing interest in the link between the clinical features of KOA and the concomitant foot and ankle symptoms^9,19^. This was confirmed by Paterson et al.^20^ who emphasized that foot pain was prevalent in these people with symptomatic KOA patients, and those with concurrent KOA and foot pain experience greater KOA–related pain and symptom severity. Meanwhile, the presence of contralateral foot/ankle symptoms, in particular, increased the risk of developing both knee symptoms and symptomatic radiographic KOA^21^. Some research has shown that LWI can increase the ankle eversion and external eversion moment whilst decreasing the external KAM effectively^11,19,22^. In addition, LWI, a widespread remedy for patients experiencing osteoarthritis in its early to mid-stage, is intended to modify the lower limb mechanical axis. Nevertheless, it should be noted that the end of the lower limb mechanical axis is the center of the ankle joint^23^ instead of encompassing the entire foot. Due to the interventional of the lateral wedge insole, it can affect foot motion, but changes in internal joint motion in particular are often overlooked. Studies on changes in foot joint motion by lateral wedge insoles are insufficient and understudied.

Based on the above, the primary objective of this study was to systematically identify the kinematic effect of the LWI on the lower limb joints by employing a combination of a lower extremity model and the right Oxford foot model (OFM). The secondary aim was to analyze the effect of LWI on the GRF and location of COP. The third purpose was to evaluate the correlation between the peak lower limb joints angles and the change of insoles. This study provides a more detailed analysis of the functions and limitations of LWI, complementing existing guidelines for the use of LWIs. The choice of LWIs for KOA patients to avoid exacerbation of existing foot symptoms seems to be critical.

## Results

### Tempo-spatial parameters

Group averages and test statistics concerning tempo-spatial parameters were presented in Table 1. The stride length, gait velocity and cadence under all conditions were consistent. The differences of these parameters among the conditions were not statistically significant (*p* > 0.05).

**Table 1:** Tempo-spatial parameters of the participants under all conditions (barefoot and wearing shoes with insoles).

Tempo-spatial Parameters	Barefoot	FI	LWI-7 mm	LWI-10 mm	*p*-Value
Stride length (m)	1.52 ± 0.05	1.57 ± 0.07	1.56 ± 0.07	1.55 ± 0.05	0.31
Gait velocity (m/s)	1.34 ± 0.13	1.42 ± 0.11	1.37 ± 0.10	1.36 ± 0.12	0.29
Cadence (steps/min)	106.32 ± 7.20	107.79 ± 5.62	105.47 ± 5.14	106.43 ± 5.32	0.89

Data were reported as mean ± SD; *p*-Value was reported as the results of repeated measure one-way ANOVA; *p* < 0.05 was considered as statistically significant.

### Lower limbs joints kinematics

**Figure 1:**
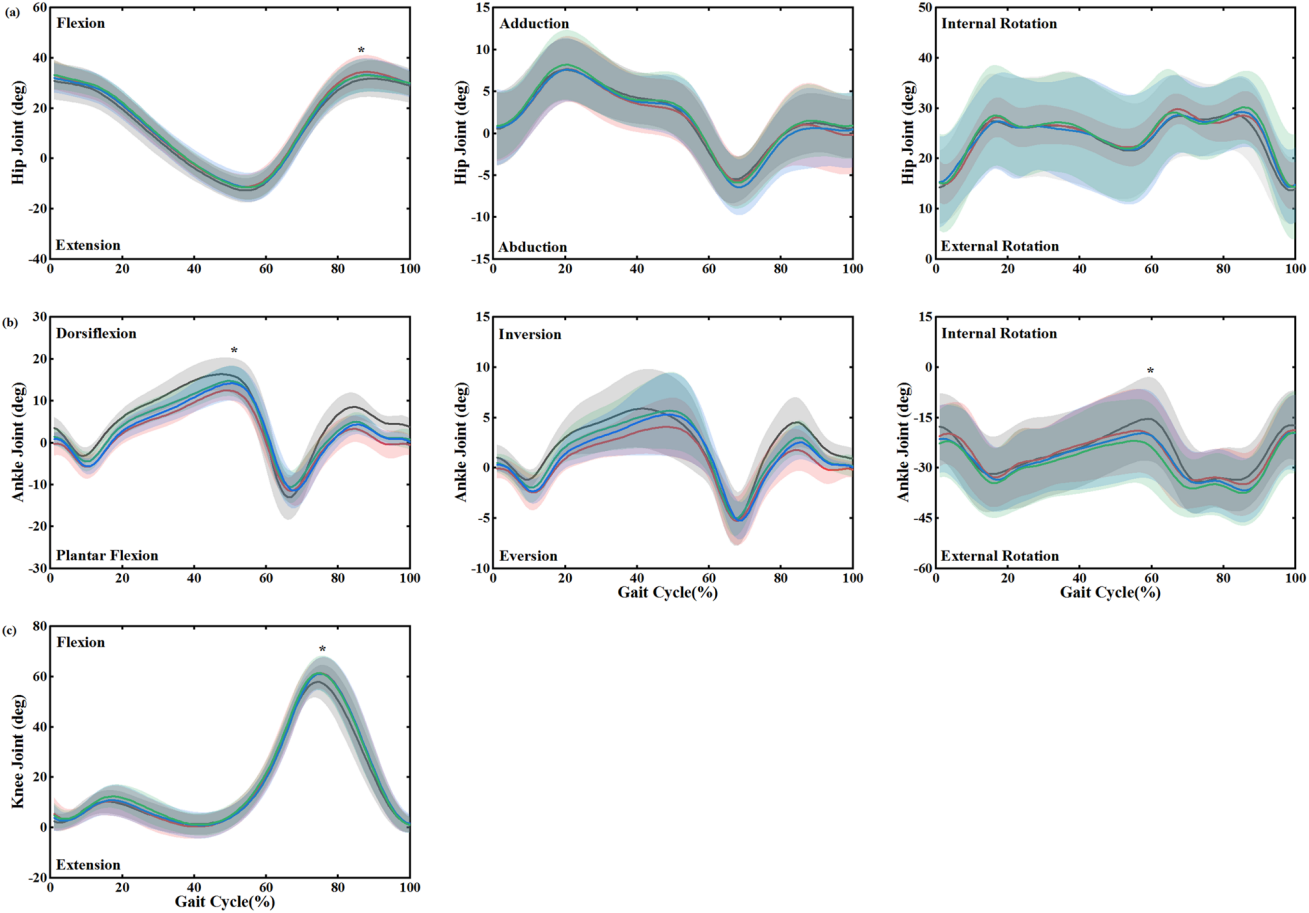
Lower limb joint angles (mean ± SD) of a gait cycle for barefoot and wearing shoes with insoles. (**a**) Hip and (**b**) ankle motion (degrees) in the sagittal, frontal, and transverse planes. (**c**) Knee joint motion (degrees) in the sagittal plane. *Indicated a significant intergroup difference in the location.

**Figure 2:**
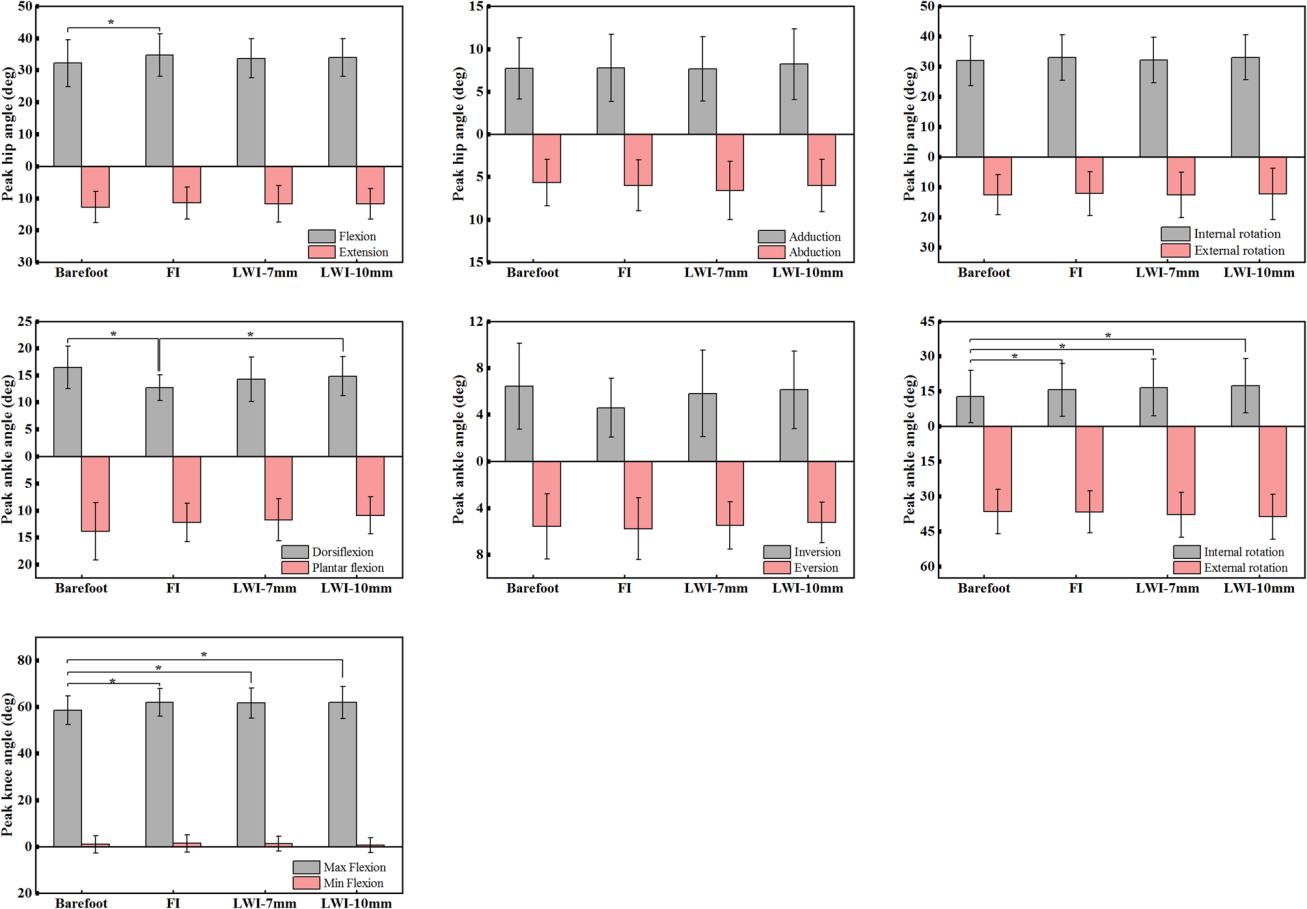
The peak values (mean ± SD) of the angles of hip, knee, and ankle joints of all participants in three planes under all conditions (barefoot and wearing shoes with insoles). *p < 0.05.

The trends of the joint angles of the hip, knee and ankle during the gait cycle in the sagittal, frontal and transverse planes were generally consistent under all conditions (Fig. 1). The analysis showed that the changes of some of the peak angles of the joints were statistically significant. (Fig. 2). For hip motion, the intervention of the insoles increased the angles of hip flexion, abduction and internal rotation (Fig. 1a). The ANOVA showed a main effect on the peak flexion for all conditions (*F*_(3,45)_ = 3.86, *p* = 0.02, *η*_*p*_^2^ = 0.355). Compared with the barefoot, post hoc analysis showed that the FI resulted in a significantly greater flexion during the terminal swing phase (*p* = 0.03) and the increment was up to 7.8%. However, other changes in the hip motion were not significantly different (*p* > 0.05).

As for the ankle angle, the ANOVA analysis showed that the peak dorsiflexion (*F*_(3,45)_ = 3.90, *p* = 0.02,* η*_*p*_^2^ = 0.358) and the peak internal rotation (*F*_(3,45)_ = 8.27, *p* < 0.01, *η*_*p*_^2^ = 0.542) were significantly affected by the insoles. Compared to the barefoot condition, the peak dorsiflexion angle was significantly decreased by 3.79°, 2.19° and 1.66° with 0, 7 and 10 mm LWIs, respectively. The largest reduction occurred in the FI, up to 23.0%. From the post hoc test, the change was statistically significant (*p* = 0.02). Compared to the FI condition, the difference between the dorsiflexion angle in the terminal stance was significant in the LWI-10 mm condition (*p* = 0.01). Compared to the barefoot condition, the internal rotation angle was increased by 2.78°, 3.76° and 4.58° with 0, 7 and 10 mm LWIs, respectively (barefoot vs FI, *p* = 0.02; barefoot vs LWI-7 mm, *p* = 0.03 and barefoot vs LWI-10 mm, *p* < 0.01).

For the knee motion, there was a significant difference in the peak flexion among all conditions (*F*_(3,45)_ = 6.75, *p* < 0.01,* η*_*p*_^2^ = 0.491). From the post hoc test, the results demonstrated the insoles of 0 mm, 7 mm and 10 mm wedges significantly increased the peak flexion angle by 3.43°, 3.09° and 3.27° respectively compared to the barefoot condition during the mid-swing phase (barefoot vs FI, *p* = 0.03; barefoot vs LWI-7 mm, *p* = 0.02 and barefoot vs LWI-10 mm, *p* = 0.01).

**Figure 3:**
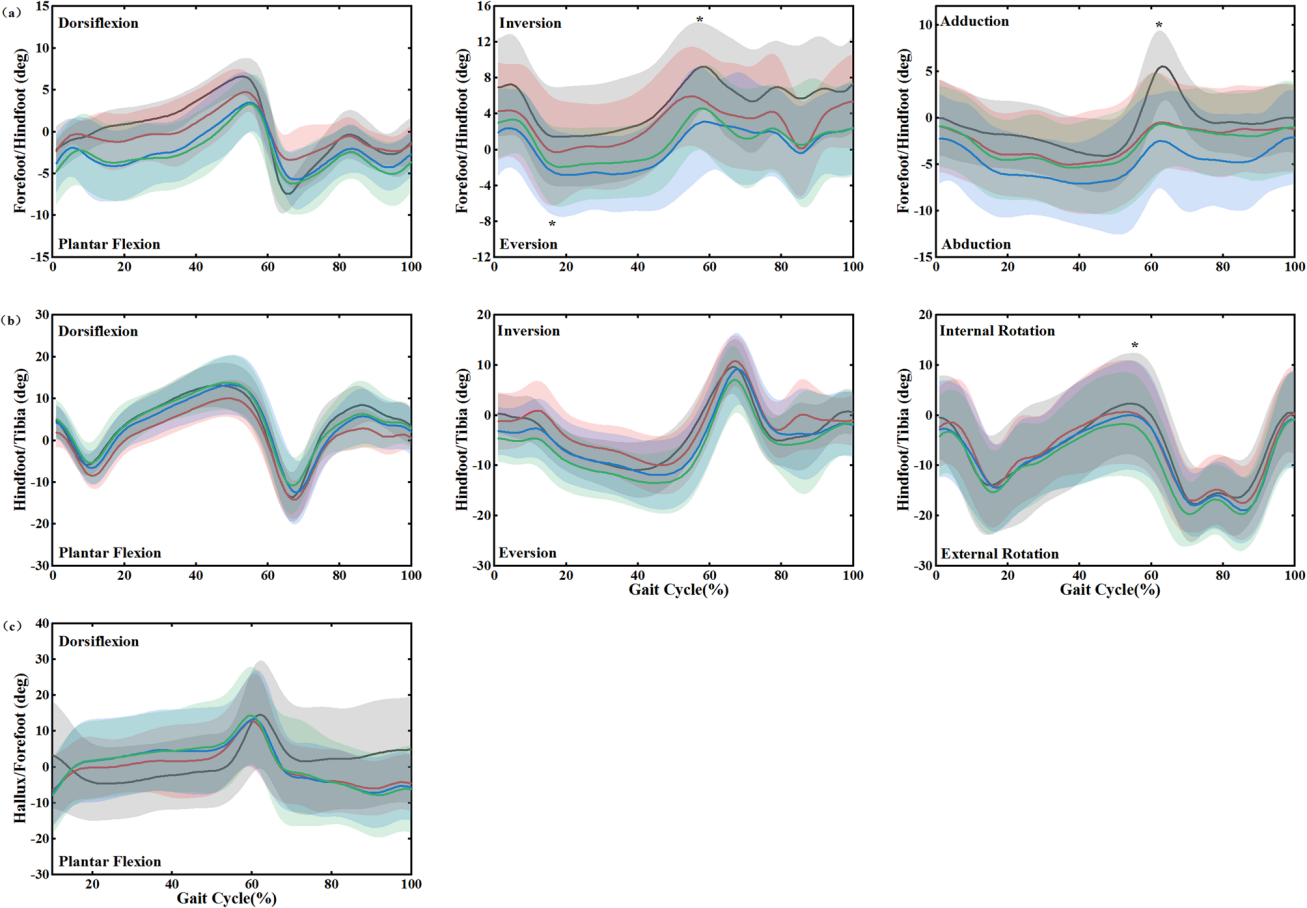
The foot kinematics (degree) in three different planes during the gait cycle for barefoot and wearing shoes with insoles. The shaded band shows mean ± SD in all controls. (**a**) Forefoot relative to hindfoot and (**b**) hindfoot relative to tibia motion (degrees) in the sagittal, frontal, and transverse planes. (**c**). Hallux relative to forefoot movement (degrees) in the sagittal plane. *Indicated a significant intergroup difference in the location.

**Figure 4:**
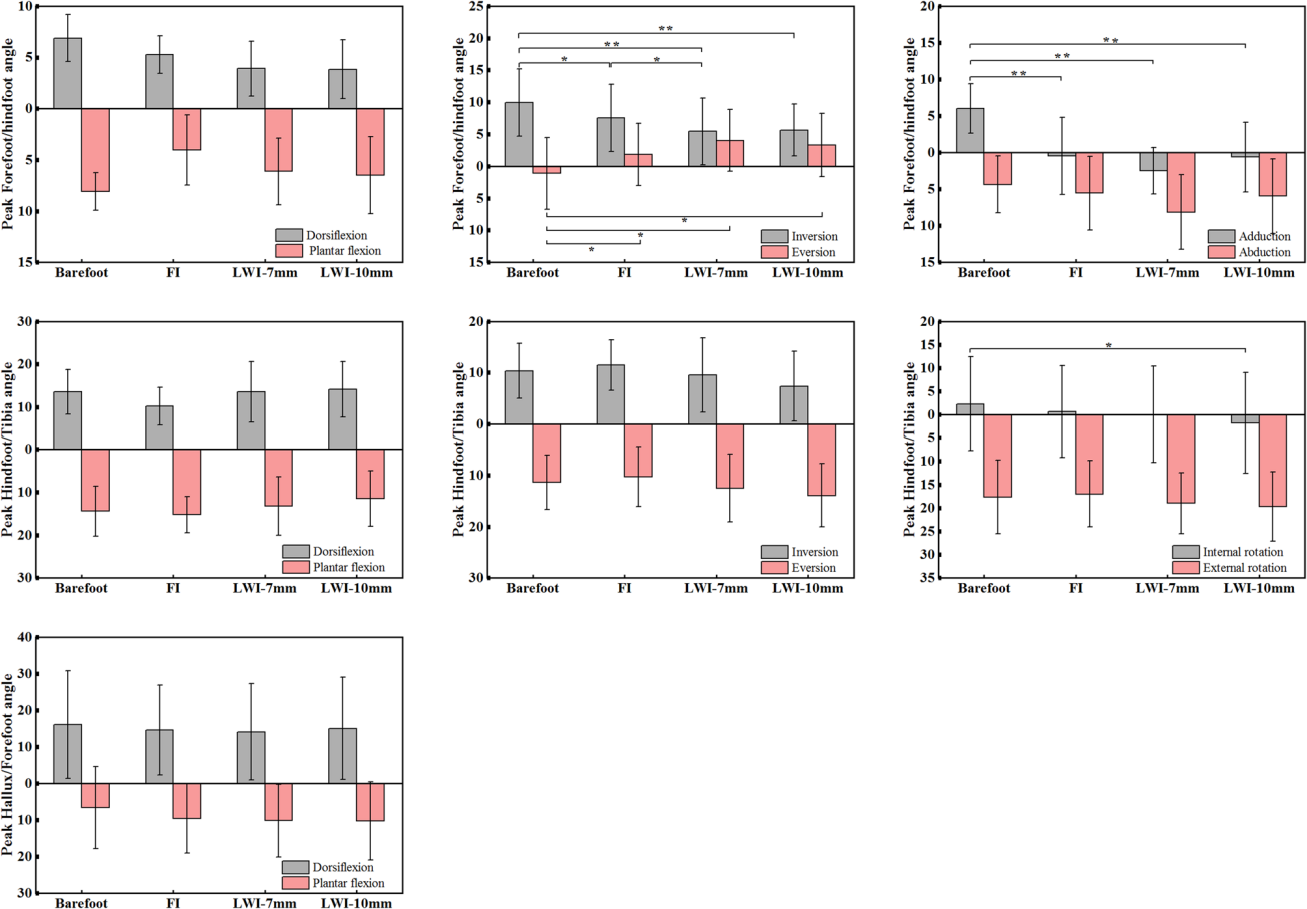
The peak values (mean ± SD) of the angle of the forefoot relative to hindfoot, hindfoot relative to tibia and hallux relative to the forefoot of all participants in three planes under all conditions (barefoot and wearing shoes with insoles). *p < 0.05.

Figure 3 showed the foot kinematics during a gait cycle and the peak angles with statistical results presented in Fig. 4. For the forefoot relative to the hindfoot in the sagittal plane, the motion angles of dorsiflexion and plantar flexion in the experimental conditions were larger than that in the control condition. However, there was no significant intragroup difference (*p* = 0.12 and *p* = 0.07). Moreover, the angles in the FI were also larger than that in the LWI-7 mm and 10 mm conditions (Fig. 3a). In the frontal plane, the peak inversion (*F*_(3,45)_ = 8.47, *p* < 0.01, *η*_*p*_^2^ = 0.548) and eversion (*F*_(3,45)_ = 6.40, *p* < 0.01, *η*_*p*_^2^ = 0.478) presented highly significant differences within groups. Post hoc analysis showed that the peak inversion and eversion angles in the experimental conditions were significantly decreased than that in the control condition during stance phase (inversion decrease: FI: 2.39°, *p* = 0.02; LWI-7 mm: 4.48°, *p* < 0.01; LWI-10 mm: 4.3°, *p* < 0.01, and eversion decrease: FI: 2.99°, *p* = 0.02; LWI-7 mm: 5.13°,* p* = 0.02; LWI-10 mm: 4.41°, *p* = 0.02). In addition, the inversion was significantly decreased by 2.02° in the LWI-7 mm condition compared to the control condition (*p* = 0.02). In the transverse plane, the peak adduction revealed an ANOVA main effect across the conditions (*F*_(3,45)_ = 11.21, *p* < 0.01, *η*_*p*_^2^ = 0.616)*.* From the post hoc analysis, the experimental conditions showed a significantly decreased adduction of 6.46°, 8.48° and 6.62° with 0, 7 and 10 mm LWIs, respectively, during the stance phase compared to the control condition.

At the hindfoot, the ANOVA analysis showed a significant difference within groups only on peak internal rotation (*F*_(3,45)_ = 3.92, *p* = 0.02, *η*_*p*_^2^ = 0.355). In the sagittal plane, the peak dorsiflexion in FI was decreased by 3.34° compared to the control condition (Fig. 3b). However, it increased slightly in the LWI-7 mm (0.01°) and LWI-10 mm (0.62°) conditions. The peak plantar flexion was increased by 0.82° in the FI, but it decreased in the LWI-7 mm (1.15°) and LWI-10 mm (2.95°) conditions. This change was the opposite of the dorsiflexion. In the frontal plane, the peak inversion in the FI was increased by 1.11° compared to the control condition at terminal stance, however, the values of the LWI-7 mm and LWI-10 mm were decreased. The peak eversion was decreased by 1.12° in the FI but increased in the LWI-7 mm and 10 mm condition at mid-stance. In the transverse plane, the hindfoot peak internal rotation was decreased by 1.67°, 2.28° and 4.07° with 0, 7 and 10 mm LWIs, respectively (*p* = 0.02). The LWI-10 mm presented a significantly decreased internal rotation compared to the control based on the post hoc analysis.

For hallux relative to forefoot movement, the ANOVA revealed that there was no significant difference in the sagittal plane during the gait cycle (*p* > 0.05). However, more importantly, the intervention of insoles presented a different trend from the control. Before the foot flat and late swing stage, the hallux was dorsiflexion barefoot, yet plantar flexion in the experimental conditions. However, during foot flat to heel off, the motion was plantar flexion barefoot, but dorsiflexion in the experimental conditions.

### Ground reaction forces and centre of pressure

**Figure 5:**
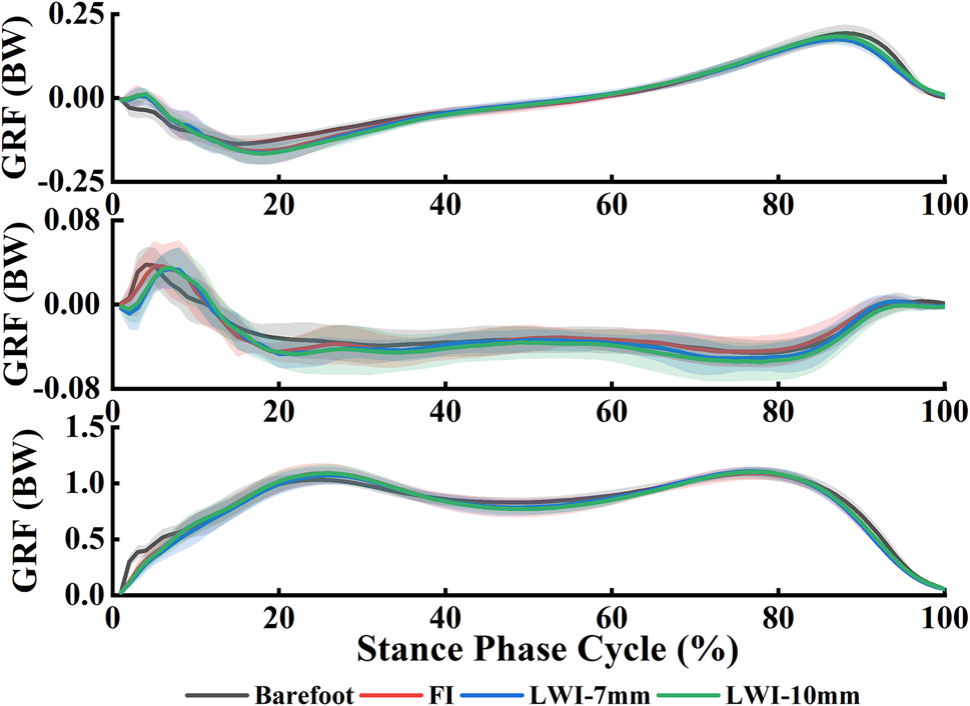
GRFs (mean ± SD) of all participants in barefoot and wearing shoes with insoles during the stance phase.

**Figure 6:**
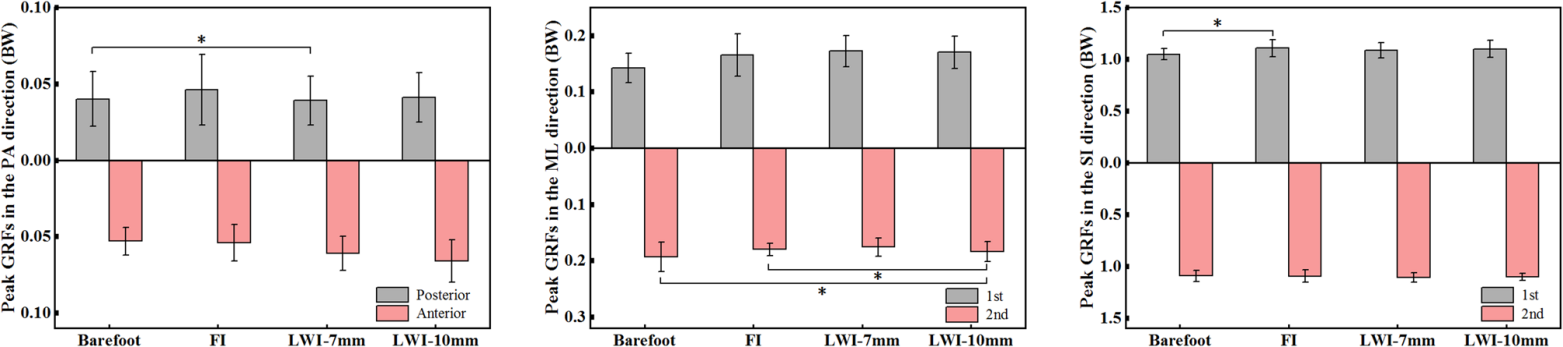
The peak GRFs (Mean ± SD) of all participants in barefoot and wearing shoes with insoles in three directions. *PA* posterior-anterior, *SI* superior-inferior, *ML* medial–lateral. *p < 0.05.

The GRFs of the 16 participants with 5 trials in the control and the experimental conditions during the stance phase was presented in Fig. 5 and all curves showed similar trends. Figure 6 showed the statistical results of GRFs of all participants in conditions of barefoot and wearing shoes with insoles in three directions.

The ANOVA showed a main effect of the experimental conditions on the peak posterior force (*F*_(3,45)_ = 3.142, *p* = 0.047, *η*_*p*_^2^ = 0.310) in the sagittal axis. The posterior force was increased in the experimental conditions compared to the control condition. From the post hoc analysis, the LWI-7 mm resulted in a significant increase of posterior force compared to the control condition. In the frontal axis, the ANOVA analysis showed that the second peak force (*F*_(3,45)_ = 5.304, *p* = 0.007, *η*_*p*_^2^ = 0.431) in the medial–lateral (ML) direction presented significant difference across all the conditions. In the post hoc test, the second peak force in the LWI-10 mm condition was significantly increased by 0.013 body weight (BW) compared to the barefoot (*p* = 0.013) and 0.012 BW (*p* = 0.002) compared to the FI. In the vertical axis, the first and second of the superior-inferior (SI) direction in the experimental conditions were higher than that in the control condition. The ANOVA analysis for the second peak forces (*F*_(3,45)_ = 4.31, *p* = 0.016, *η*_*p*_^2^ = 0.381) showed a significant difference across all the conditions. Post hoc analysis showed that the first peak force in FI was increased by 0.06 BW compared to the control condition (*p* = 0.027).

**Figure 7:**
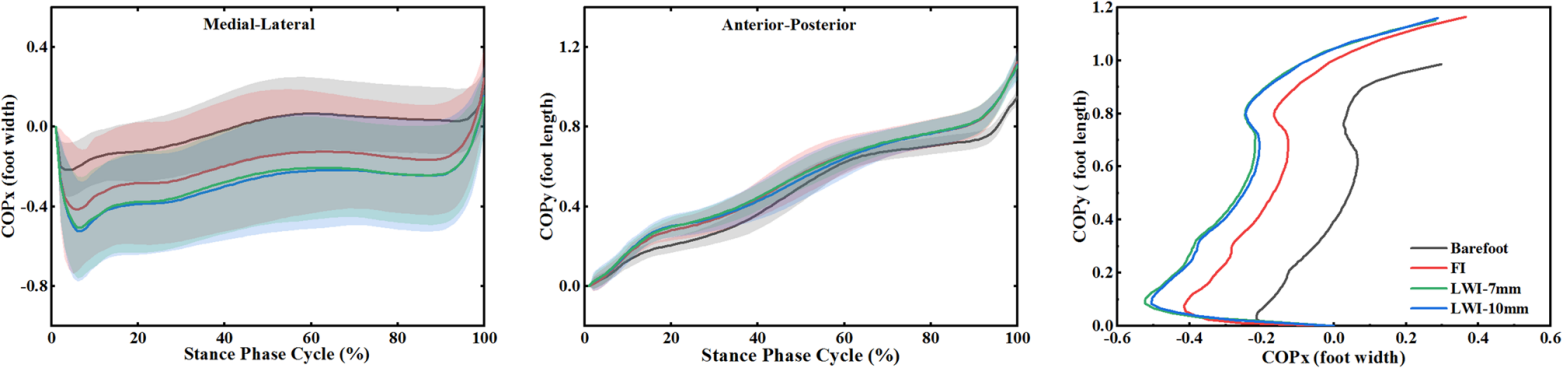
COP_x_ (left), COP_y_ (medium), and COP in the transverse planes (right) of subjects in barefoot and wearing shoes with insoles conditions during the stance phase.

Figure 7 showed the location of the COP during the stance phase for subjects barefoot and for wearing shoes with insoles. The intervention of insoles made the location of COP shift laterally. The FI condition made the COP move laterally about 20% of feet width. The LWI-7 mm and LWI-10 mm conditions resulted in a similar outward deviation, however, the COP shift is more than that in the FI condition, about 30% of feet width.

### Correlation analysis of insoles and lower limb joint angles

Spearman correlation analysis was applied to determine the relationship between the change of insoles and the angle of lower limb joints (Table 2).

**Table 2:** Spearman correlation coefficients (r) between the peak joint angles and the conditions changing from barefoot to flat insole; between the peak joint angles and the conditions changing from FI to LWI-7 mm and LWI-10 mm.

Peak joint angle	Conditions changing from Barefoot to FI r (*p*)	Conditions changing from FI to LWI-7 mm and LWI-10 mm r (*p*)
Hip joint angle		
Peak flexion	0.194 (0.079)	− 0.047 (0.613)
Peak extension	− 0.117 (0.294)	− 0.018 (0.845)
Peak adduction	− 0.044 (0.691)	0.059 (0.525)
Peak abduction	0.014 (0.899)	− 0.073 (0.434)
Peak internal rotation	0.031 (0.780)	0.003 (0.977)
Peak external rotation	− 0.103 (0.356)	0.035 (0.704)
Knee joint angle		
Max flexion	0.200 (0.070)	0.036 (0.703)
Min flexion	− 0.013 (0.907)	0.098 (0.295)
Ankle joint angle		
Peak dorsiflexion	− 0.599 (< 0.001)**	0.273 (0.003)**
Peak plantar flexion	− 0.102 (0.361)	− 0.179 (0.054)
Peak inversion	− 0.310 (0.004)**	0.201 (0.030)*
Peak eversion	0.066 (0.551)	− 0.096 (0.302)
Peak internal rotation	− 0.103 (0.356)	− 0.062 (0.510)
Peak external rotation	− 0.036 (0.745)	0.091 (0.331)
Forefoot relative to hindfoot		
Peak dorsiflexion	− 0.372 (0.001)**	− 0.090 (0.336)
Peak plantar flexion	− 0.427 (< 0.001)**	0.196 (0.034)*
Peak inversion	− 0.213 (0.053)	− 0.210 (0.023)*
Peak eversion	− 0.264 (0.016)*	− 0.177 (0.056)
Peak adduction	− 0.533 (< 0.001)**	− 0.031 (0.740)
Peak abduction	− 0.173 (0.118)	− 0.037 (0.690)
Hindfoot relative to the tibia		
Peak dorsiflexion	− 0.295 (0.007)**	0.298 (0.001)**
Peak plantar flexion	0.073 (0.509)	− 0.214 (0.021)*
Peak inversion	0.057 (0.607)	− 0.127 (0.173)
Peak eversion	− 0.062 (0.575)	0.173 (0.062)
Peak internal rotation	− 0.023 (0.836)	− 0.076 (0.418)
Peak external rotation	− 0.080 (0.470)	0.130 (0.161)
Hallux relative to the forefoot		
Peak dorsiflexion	− 0.029 (0.793)	− 0.037 (0.695)
Peak plantar flexion	− 0.117 (0.294)	− 0.142 (0.127)

*Statistically significant correlation (*p* < 0.05).

**Highly statistically significant correlation (*p* < 0.01).

Spearman correlation coefficients with and without flat insoles were used to investigate the correlation between the intervention of insole and lower limb joints (Table 2). For the ankle, the peak dorsiflexion (r = − 0.599, *p* < 0.001) and inversion (r = − 0.310, *p* = 0.004) were significantly negatively correlated with the intervention of insole. Focusing on forefoot joints, the peak dorsiflexion (r = − 0.372, *p* = 0.001), plantar flexion (r = − 0.427, *p* < 0.001) and adduction (r = − 0.533, *p* < 0.001) showed a highly significant negative correlation with the intervention of insoles. In addition, the hindfoot dorsiflexion and intervention of insoles had a significant negative correlation (r = − 0.295, *p* = 0.007).

Spearman correlation coefficients FI, LWI-7mm and LWI-10mm conditions were used to investigate the correlation between wedge height and lower limb joints. The variables showed a correlation between wedge height and the ankle, forefoot and hindfoot angles. For the ankle, there were a significant positive correlation between wedge height and the peak dorsiflexion (r = 0.273, *p* = 0.003) as well as inversion (r = 0.201, *p* = 0.030). As for the forefoot, the peak plantar flexion (r = 0.196, *p* = 0.034) showed a significant positive correlation with the wedge height. Yet, the peak inversion (r = − 0.210, *p* = 0.023) showed a significantly negative correlation with wedge height. In addition, the hindfoot joints in the sagittal plane (dorsiflexion: *p* = 0.001; plantar flexion: *p* = 0.021) and wedge height showed a significant correlation. The peak dorsiflexion (r = 0.298) had positive correlations with wedge height, but the peak plantar flexion (r = − 0.214) was negatively correlated with wedge height.

## Discussion

In the present work, the experimental measurements were adopted to investigate the effects of LWI at normal walking speeds on the biomechanics of the lower limb joints during the gait cycle, including lower-limb joint kinematics, GRFs, and COP. The right OFM can not only reflect the motion changes of the hip, knee, and ankle but also analyze the movement of the foot in detail. The results highlighted that the intervention of LWI does indeed influence these values, especially on the knee and ankle joint angles, as well as the forefoot and hindfoot in foot kinematics. In addition, the distribution of GRFs and COP was altered. All the above parameters showed significant differences in certain planes in the intragroup comparison.

The LWI affected the hip (*p* = 0.02), knee (*p* < 0.01) and ankle (*p* = 0.02) joint angles in the sagittal plane, as shown in Fig. 2. It was found that the increase in the flexion angles of the knee and hip was accompanied by a decrease in the ankle dorsiflexion angle due to the intervention of the insoles, as shown in Fig. 1. With increasing wedge height, the flexion angle of the knee and hip becomes larger whilst the dorsiflexion angle of the ankle decreases. The results on the ankle were previously demonstrated by Russell et al.^24^. However, our work was mainly focused on the coordination and interaction of the lower extremity joints during human movement. The LWIs are closer to the ankle than the hip and knee, so ankle motion is more susceptible to the LWI as demonstrated by the reduced range of motion in this study. In addition, the study also found that the internal rotation angle of the ankle increased because of the insoles, and the larger the wedge height difference, the wider the internal rotation angle (Fig. 2).

The findings of this study showed a significant difference in the motion of the forefoot in the movement planes, and the hindfoot in the frontal plane when participants were barefoot and wearing shoes with LWI, as shown in Fig. 4. The right OFM is a combination of the lower limbs model and the OFM. The OFM defined by Carson et al.^25^ was an effective method that was widely used in clinical testing and motor rehabilitation, and its repeatability has been demonstrated^26,27^. The trends of the curves of the forefoot in the present study were very similar to the previous gait research results. Zhang et al.^28^ concluded that the amplitude and variability of the forefoot relative to the hindfoot would not be significant due to the specificity of its anatomical structure. The results of our study were different. A wedge height of 7 mm and 10 mm was designed to create a 5-degree and 7-degree valgus foot. Figure 3a showed the valgus caused a more persistent period of plantar flexion and eversion. During the transition from the stance phase to the swing phase, the movement of the forefoot was completely restricted to three planes due to the addition of insoles. The range of motion of the forefoot joint was passively reduced by the LWI, except for the plantar flexion. The long-term limitation of motion caused a corresponding decrease in muscle tone, muscle atrophy, and reduced extension and flexibility of the foot muscles in patients with lateral knee osteoarthritis. Poor muscle strength makes it difficult to maintain the stability of the joints, allowing the joints to move beyond their range of motion, which ultimately tends to lead to joint injuries in the foot.

The hindfoot is the distal connecting part of the lower limb joint, and motion at the talocrural and subtalar joints are considered to contribute jointly to the motion of the hindfoot relative to the tibia^25^. Some studies^29^ demonstrated that the coupling motion between the subtalar joint and the leg altered hindfoot motion^30^ may affect tibial motion which may consequently affect the function of proximal joints, such as the knee^31^. Our study also obtained the same results. As shown in Fig. 3b, it can be found that the eversion angle of the talocrural joint increased under the action of the LWI, which was accompanied by an increase in the internal rotation angle of the shank and femur in the lower limb joint angle. In addition, Chapman et al^19^ proved that someone with a greater everted ankle/subtalar complex (i.e., the foot being modeled as a rigid and single segment) under control conditions was more likely to have a decrease in peak KAM with the LWI^32^. Thus, this study also provided some indirect evidence that LWI may reduce external KAM. However, it is also not to be underestimated that the increased eversion caused by insoles may also have the potential to trigger some foot diseases. It increases the tension on the medial ankle and the pressure on the lateral ankle, which will lengthen and weaken the medial collateral ligament, making the medial ankle prone to sprains.

The effect of the LWI on the kinematics of the lower limb joints is obvious, but the fact is that it is a direct alteration of the GRFs and the COP. Mohamad Hosein Ghasemi's study^33^ focused on evaluating in detail the COP of external wedge insoles during weight lifting. However, the position of our COP was mobile during walking. In this study, the GRFs showed an effect of the intervention of LWIs which increased the vertical GRF and braking force (the posterior component of the GRF vector) (Fig. 5). For normal adults walking, a change in the peak vertical GRF is accompanied by a change in the horizontal component GRF (braking GRF and propulsive GRF)^34^. The change of vertical GRF affects the external KAM. In addition, it was observed that the COP shifted laterally by the application of LWI as expected from theoretical biomechanical assumptions^9^ and a previous study by Kakihana et al^7^. However, the two different wedge heights have little effect on the degree of COP deviation. A study^35^ explained the mechanism of the reduction of KAM by LWI is due to a lateral shift in foot COP and a consequent shortening of the knee lever arm (KLA). KAM can be simply calculated as KLA multiplied by the frontal plane ground reaction force (FP-GRF). Therefore, the increase in the vertical GRF and the lateral shift of COP should be adopted as factors affecting KAM.

Meanwhile, the correlation analysis showed a correlation between the intervention of insoles and some angles of the lower limb joints. In this study, the ankle, forefoot, and hindfoot angles in the sagittal plane were significantly in correlation with the LWIs. The presence of the insole and wedges can significantly affect the range of motion in the foot and has an impact on foot health. Rehabilitation therapists should consider foot health when using LWI to delay early knee osteoarthritis in the clinical setting^36^, especially to prevent foot and ankle joint disease. It is particularly important to develop patient-specific LWI insoles.

In this study, the relationship between the joints of the human lower extremity and the foot insoles was considered during walking. The changing pattern of their motion, GRFs, and COP were systematically described and summarized in detail for the LWI. However, there are still many limitations to the study. Firstly, healthy college students were selected as the samples but knee osteoarthritis occurs mostly in the elderly, thus the results should be further investigated if they are similar in older patients with knee osteoarthritis. Secondly, the study adopted a combination of the lower limb model and the OFM. The midfoot segment of OFM only functions as a mechanism transmitting motion of the forefoot and hindfoot, and its real role has not been effectively discussed. If we want to study the real motion changes of the foot joints in detail, more detailed foot kinematic models need to be introduced. Thirdly, the same insoles were used by all participants, but the effect of the shoes on the results has not been considered. Finally, the study did not take the types of foot alignments into account, the number of subjects may not be completely enough.

## Conclusion

The study combined a lower extremity model with the OFM to provide a detailed statistical analysis of the effects of LWI on the lower extremity joints, especially on the foot joints, GRFs, and COP during a gait cycle. It was found that the LWI increased the hip and knee motion but decreased ankle motion in the sagittal plane. Moreover, forefoot movement was restricted and hindfoot eversion was increased. The GRFs were changed and the COP was shifted laterally by the application of LWI. These changes should be taken into consideration by clinicians, researchers, and related rehabilitation specialists to prevent medial ankle sprains and other foot and ankle disorders while treating knee osteoarthritis with LWI.

## Materials and methods

### Participants

It was not easy for us to recruit KOA patients, other researchers^37,38^ have used healthy individuals to study the effects of LWIs, therefore we conducted the study with healthy individuals. Estimation of the sample size was performed using the Gpower software (3.1 software, Düsseldorf, Germany) applying ANOVA repeated measures (F Test) with a power of 0.9, at a 5% significance level, and an effect size of 0.25. The sample size of 16 subjects were generated. 16 healthy participants (6 males and 10 females, age 26 ± 2 years old, weight 58.55 ± 11.99 kg, height 1.67 ± 0.08 m, and BMI 20.86 ± 2.90 kg/m^2^) were recruited from the Taiyuan University of Technology in this study. In addition, all participants reported having no history of heart or respiratory disease or an uncorrected visual impairment, no previous lower extremity fracture or surgery, and having normal ankle function; all subjects had no experience of using related orthopaedic insoles in the past three months. An informed consent was obtained from all participants. All methods were carried out in accordance with relevant guidelines and regulations. The study was conducted in accordance with the Declaration of Helsinki, and the protocol was approved by the University Research Ethics Committee, Taiyuan University of Technology (TYUT202105001).

### Equipment and procedure

**Figure 8:**
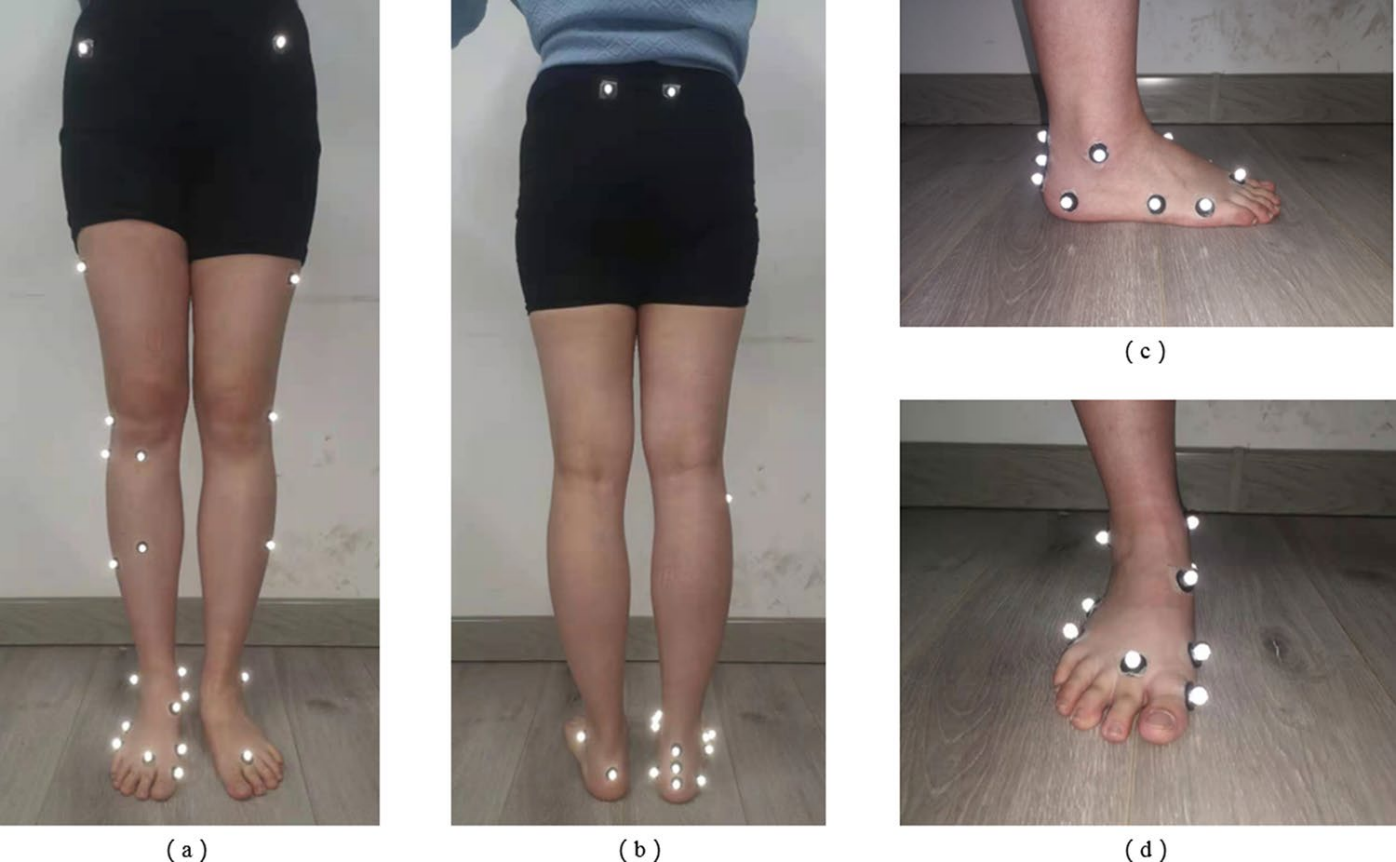
The right Oxford foot model and the placement of the markers. (**a**) Front view. (**b**) Rear view. (**c**) Foot marker placement details from the right side view. (**d**) Foot marker placement details from the front side view.

Before the experiment, each participant's height, weight, leg length, knee width, and ankle width were measured to build the model in the Vicon system. The kinematics for all participants were captured and recorded by using the Vicon motion analysis system (Vicon MX, Vicon Motion System Ltd., Oxford, England) at the frequency of 100 Hz. The system was synchronized with two centrally embedded force plates (Kistler, type 9865B, Winterthur, Switzerland and AMTI, OR6, USA, 1000 Hz) and positioned in series for capturing the location of COP, GRFs, and identifying gait cycle events. The force plates were placed in consecutive order and flush with the ground. Both plates were covered with the same walkway surface material. The Vicon Nexus software synchronously processed and analyzed the marker trajectories and force platform data. To ensure the accuracy of the experiment, all participants adopted the same relaxed standing position for 5 s, before conducting a static trial. 29 light-reflecting tracking markers of 9.5-mm diameter were matched with anatomical landmarks of the right OFM^25^. They were directly attached to the skin using double-sided tape. The specific position of the markers (Fig. 8) were placed as close as possible to the correct anatomical landmarks of the human body with the help of physicians, referring to the Vicon instruction manual. After collecting the required static trials, three markers were removed from the right leg before a dynamic trial.

**Figure 9:**
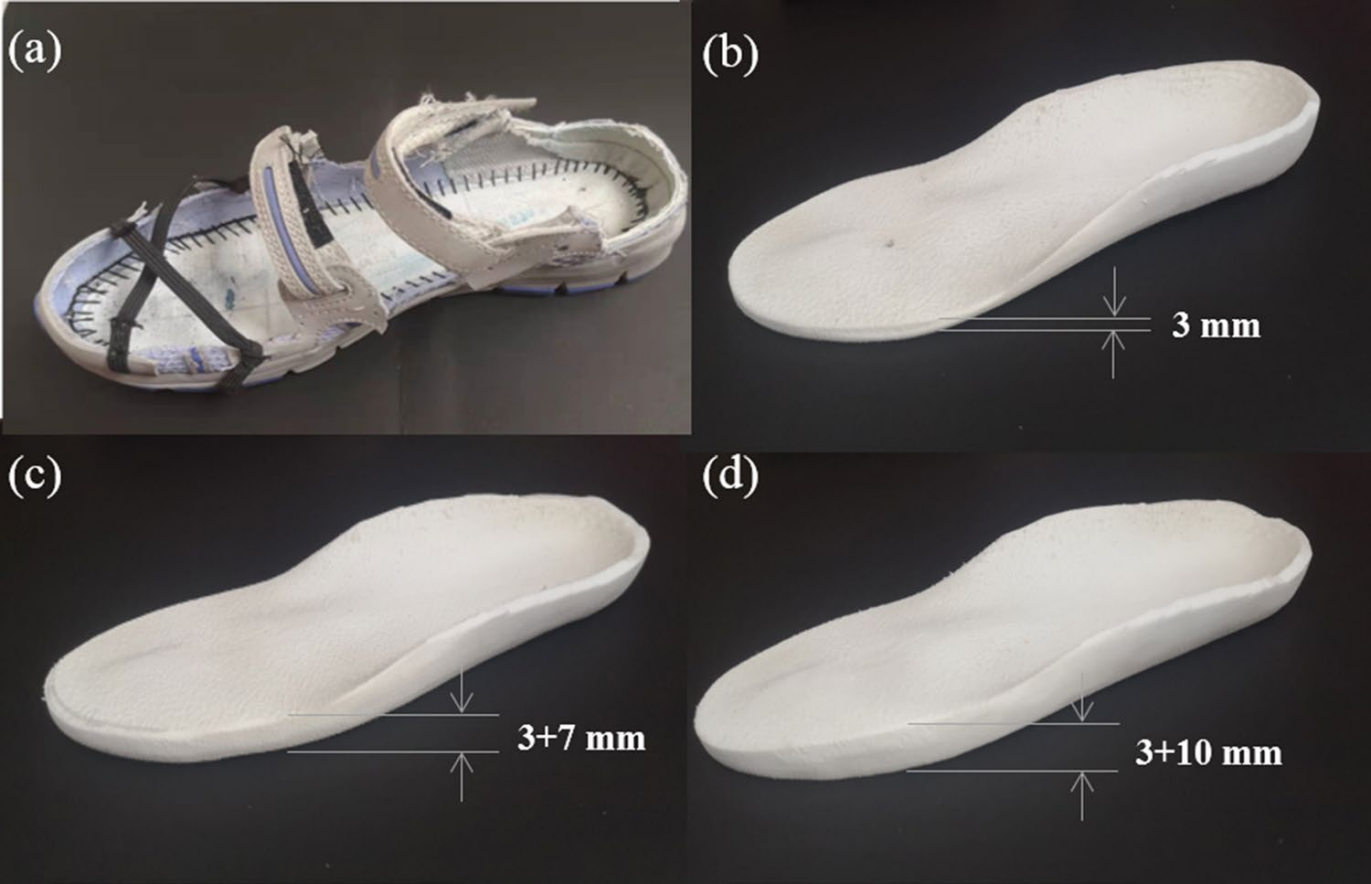
(**a**) An assigned lab shoe. (**b**) FI. (**c**) LWI-7 mm. (**d**) LWI-10 mm.

For the experiment, barefoot gait was designed as the control condition, and wearing assigned lab shoes (Fig. 9a) with custom LWIs was used as the experimental condition. The shoes (sandals) with straps that allow the placement of markers on the forefoot and hindfoot were used for each experimental condition. Insoles were inserted in the lab shoes bilaterally without removing the markers. All insoles were customized by Shanxi Rongjun Rehabilitation Auxiliary Centre and made of ethyl vinyl acetate (EVA) foam, the stiffness is 30° A Shore. The EVA material has a Young’s modulus of 84 MPa, yield stress of 10 MPa and density of 940 kg/m^3^. The 2D plantar pressure plate and 3D plantar feature scanner were operated to confirm the static and dynamic foot pressure distribution parameters of the subject. The orthopaedic insole production system (GEBIOM, Germany) together with the auxiliary design software (GP Manager) was used to complete the customized insoles. Later, arch supports were equipped at the horizontal and vertical arches to improve wearing comfort. The experimental condition details are described as follows:

1. Control condition: barefoot;
2. Experimental conditions: wearing a pair of assigned lab shoes (Fig. 9a) with three types of insoles. They were:

a) Flat insoles (FI), an insole with 0 mm lateral wedge and 3 mm base thickness (Fig. 9b),

b) 7 mm lateral wedge insole (LWI-7mm) which is based on the flat insoles and could create a 5-degree valgus foot (Fig. 9c),

c) 10 mm lateral wedge insole (LWI-10 mm) which is based on the flat insoles and could create a 7-degree valgus foot (Fig. 9d).

A cross-over randomized design was used for each participant during the experiment. All participants needed to walk several times to adapt to the whole experimental set-up and conditions before formal data collection. Then they were required to walk through an experimental walkway, about 10 m in length, at a self-selected normal speed, booth barefoot and wearing insoles. Gait analysis was repeated until 5 clean strikes on the force plates from the right foot could be obtained to ensure gait stability and to reduce experimental data collection error. The statistical analysis was based on the test data of the right leg.

### Data-analysis and statistics

Tempo-spatial parameters including stride length, cadence, and gait velocity were calculated. The experimental parameters were mainly lower-limb joint angles (the hip and ankle joint in the sagittal, frontal, and transverse plane; the knee joint in the sagittal plane; the hallux relative to the forefoot, the forefoot relative to hindfoot and the hindfoot relative to the tibia in the three anatomical planes), GRFs and COP during the gait cycle, including the stance phase and swing phase. The GRFs were normalized to body weight. The COP_x_ data was normalized to foot width and the COP_y_ data was normalized to the foot length. In this study, the COP shift was defined as the distance of the COP from the line of the foot (calcaneal tuberosity to the midpoint between the first and fifth metatarsal heads) where negative values of COP_x_ indicated the lateral side of the body and positive values indicated the medial side of the body. All characteristic parameters (peak joint angles and GRFs) were expressed as mean ± standard deviation (SD).

Shapiro–Wilk tests were used to check the data for normal distribution, while homogeneity of variance was investigated with Levene’s test. Boxplots were performed to check the data for outliers. Comparison of the mean values between the groups was performed using the one-way repeated measure of ANOVA. Intergroup comparison was performed by using the Bonferroni post hoc test. *p* < 0.05 was considered statistically significant and the effect size estimates were computed using partial eta squared (*η*_*p*_^2^). Spearman correlation analysis was used to investigate the correlation between the change of insoles and the peak lower limb joint angles. Statistical analyses were performed using the software SPSS Statistics 26 (SPSS Inc., Chicago, IL, USA).

## Data Availability

The data are available from the corresponding author on reasonable request.
